# Effectiveness and safety of Liuhe Pill for treating gout: A protocol for a systematic review and meta-analysis

**DOI:** 10.1097/MD.0000000000025533

**Published:** 2021-04-23

**Authors:** Chunsheng Yang, Zulifeiya Aletengbieke, Li Liu, Banu Bakeer

**Affiliations:** Department of Rehabilitation Medicine, Xinjiang Medical University Affiliated First Hospital, China.

**Keywords:** gout, Liuhe Pill, meta-analysis, prognosis, systematic review, traditional Chinese medicine

## Abstract

**Background:**

Liuhe Pill as a famous traditional Chinese medicine formula is usually used to treat gout, acute pancreatitis, and cellulitis in China. But at present, there is no systematic evaluation report on its therapeutic effectiveness and safety of Liuhe Pill for treating gout. This protocol aims to assess the efficacy and safety of Liuhe Pill for treating gout.

**Methods:**

We will search the Web of Knowledge, EMBASEWANFANG DATA, China National Knowledge Infrastructure (CNKI), PubMed, ClinicalTrials.gov and Cochrane Library from inception to October 31, 2021 to retrieve relevant studies. We will also search citations of relevant primary and review. Authors of abstract in the meeting will be further searched in PubMed for potential full articles. To minimize the risk of publication bias, we will conduct a comprehensive search that included strategies to find published and unpublished studies. Two authors independently judged study eligibility and extracted data. Heterogeneity will be examined by computing the Q statistic and *I*^*2*^ statistic.

**Results:**

This study assessed the efficiency and safety of Liuhe Pill for treating gout.

**Conclusions:**

This study will provide reliable evidence-based evidence for the clinical application of Liuhe Pill for treating gout.

## 1 Introduction

Gout is an inflammatory arthritis resulting from elevated body uric acid pools. And it is the most common form of inflammatory arthritis, affecting 9.2 million adults (3.9%) in the United States.^[1]^ Despite urate lowering therapy is recommended, adherence to ULT remains poor,^[2,3]^ the lowest among 7 common chronic medical conditions.^[4]^ Therefore, it is necessary to improve the managements of patients with gout. Previous researches showed that Liuhe Pill can reduce the inflammatory index and it has been used to treat gout in China for many years. Despite this, there are no published systematic reviews and meta-analyses exploring the effectiveness and safety of Liuhe Pill treating gout. Therefore, we conducted this systematic review and meta-analysis to clarify the efficacy and safety of Liuhe Pill for treating gout.

## 2 Methods and analysis

### 2.1 Registration

This protocol of systematic review and meta-analysis is based on the Preferred Reporting Items for Systematic Reviews and meta-analysis Protocols (PRISMA-P) statement guidelines. And the protocol has been registered on International Prospective Register of Systematic Reviews database. The registration number was INPLASY202130019.

### 2.2 Eligibility criteria

The inclusion criteria for the study will include:

1. studies with adult patients who has a diagnosis of gout;
2. conference abstracts were only included when they provided adequate relevant information for assessment;
3. the patients with gout were divided into 2 groups (treated with Liuhe Pill or without Liuhe Pill).

Exclusion criteria will include: age <18 years old, and patients with incomplete data.

### 2.3 Searching strategy

**Figure 1:**
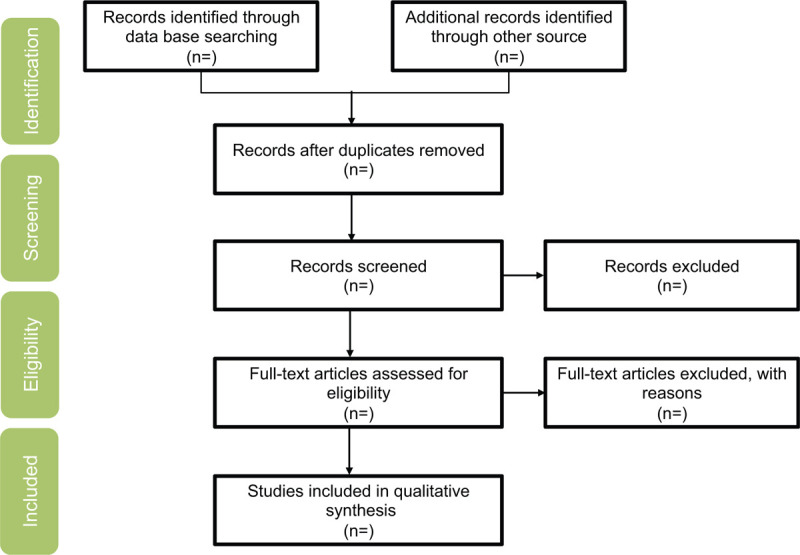
A flow diagram demonstrating the search strategy and study selection process for this study.

We will search the Web of Knowledge, EMBASEWANFANG DATA, CNKI, PubMed, ClinicalTrials.gov, and Cochrane Library from inception to October 31, 2021 to retrieve relevant studies using the search strategy: (“Liuhe Pill” OR “Liuhedan” OR “Liu-He-Dan”) AND (“gout” OR “hyperuricemia”). No language restrictions will be applied. We will also search citations of relevant primary and review. Authors of abstract in the meeting will be further searched in PubMed for potential full articles. To minimize the risk of publication bias, we will conduct a comprehensive search that included strategies to find published and unpublished studies. The research summary of the screening flow chart is shown in Figure 1.

### 2.4 Data extraction and risk of bias

Two reviewers will be employed the searching strategy respectively, by reading the papers and scoring them according to the QUADAS-2 checklist^[5]^ and Newcastle–Ottawa Quality Assessment Scale^[6]^; disagreement will be settled by a third opinion. Important information will be abstracted from the included articles in a standardized form by 2 reviewers. Important information includes the name of the first author, publication year, publication country, type of study, study population, sample size, using of Liuhe Pill, and outcomes studied (duration of pain and the proportion of subjects achieving the target serum urate level at month 6). Risk of bias assessment will be carried out according to the Newcastle–Ottawa Scale (NOS) to rate the internal validity of the individual studies, and funnel plots will be constructed to assess the risk of publication bias.

### 2.5 Statistical analysis

All pairwise meta-analytic calculations will be performed with Review Manager software (RevMan) version 5.3 (Cochrane Collaboration). Heterogeneity will be examined by computing the Q statistic and *I*^*2*^ statistic, and presence of reporting bias by visual inspection of funnel plots. Statistical significance was considered when the *P* value < .05.

## 3 Discussion

In western high-income countries, the prevalence of gout is 3% to 6% in men and 1% to 3% in women. In 2015 and 2016, the incidence of gout was 3.9% among US adults (2.7% in women and 5.2% in men).^[1]^ Gout is an inflammatory arthritis resulting from elevated body uric acid pools. Despite international specialty society guidelines^[7–10]^ recommend treat-to-target strategies with use of urate lowering therapy (ULT), over the past 2 decades there has been no increase in ULT utilization. Adherence to ULT remains poor,^[2,3]^ the lowest among 7 common chronic medical conditions.^[4]^

Liuhedan is a famous traditional Chinese medicine (TCM) formula containing Radix et Rhizoma Rhei, Cortes Phellodendri, Rhizoma Atractylodis Macrocephal, Radix Angelicae Dahur Cae, Fructus Mume, Herba Menthae, and Mel, etc.^[11,12]^ Previous researches showed that Liuhe Pill could reduce the inflammatory index by anti-inflammatory and immunomodulating and it is used to treat gout for many years in China. However, the conclusion that Liuhe Pill improve the prognosis (duration of pain and the proportion of subjects achieving the target serum urate level at month 6) of patients with gout, is controversial and it is not be adopted by other country. Therefore, we designed the systematic review and meta-analysis protocol by using the latest data to test the effectiveness and safety of Liuhe Pill in treating gout. The results of our review will be reported strictly following the PRISMA criteria.

## Acknowledgments

The authors would like to acknowledge the participants and their families for taking part in the study.

## Author contributions

**Conceptualization:** Chunsheng Yang, Li Liu, Banu Bakeer.

**Data curation:** Chunsheng Yang, Zulifeiya Aletengbieke, Banu Bakeer.

**Formal analysis:** Chunsheng Yang, Li Liu.

**Funding acquisition:** Banu Bakeer.

**Investigation:** Chunsheng Yang, Zulifeiya Aletengbieke.

**Methodology:** Chunsheng Yang, Zulifeiya Aletengbieke.

**Project administration:** Chunsheng Yang, Li Liu, Banu Bakeer.

**Resources:** Chunsheng Yang, Zulifeiya Aletengbieke.

**Software:** Chunsheng Yang, Zulifeiya Aletengbieke, Li Liu.

**Supervision:** Chunsheng Yang, Banu Bakeer.

**Validation:** Chunsheng Yang, Zulifeiya Aletengbieke.

**Visualization:** Chunsheng Yang, Li Liu, Banu Bakeer.

**Writing – original draft:** Chunsheng Yang, Zulifeiya Aletengbieke, Li Liu.

**Writing – review & editing:** Chunsheng Yang, Zulifeiya Aletengbieke, Banu Bakeer.
